# Identification and Validation of Pyroptosis-Associated Gene Signature in Primary Sjögren's Syndrome

**DOI:** 10.1155/mi/1538054

**Published:** 2025-10-03

**Authors:** Yijun Dai, Hanzhi Chen, Meng Zhou, Chenmin Wu, Fei Gao, Yingzheng Wang, Qing Yan, Zuoan Li

**Affiliations:** ^1^Department of Rheumatology and Immunology, Shengli Clinical Medical College of Fujian Medical University, Fujian Provincial Hospital, Fuzhou University Affiliated Provincial Hospital, Fuzhou 350001, China; ^2^College of Pharmacology, Fujian University of Traditional Chinese Medicine, Fuzhou 350122, China; ^3^Department of Emergency Surgery, Shengli Clinical Medical College of Fujian Medical University, Fujian Provincial Hospital, Fuzhou University Affiliated Provincial Hospital, Fuzhou 350001, China; ^4^Fujian Provincial Key Laboratory of Emergency Medicine, Fuzhou University Affiliated Provincial Hospital, Fuzhou 350001, China; ^5^Fujian Provincial Institute of Emergency Medicine, Fuzhou University Affiliated Provincial Hospital, Fuzhou 350001, China; ^6^Fujian Emergency Medical Center, Fuzhou University Affiliated Provincial Hospital, Fuzhou 350001, China

**Keywords:** bioinformatic analysis, gene signatures, primary Sjögren's syndrome (pSS), pyroptosis

## Abstract

**Background:**

Pyroptosis, a form of programmed cell death, has been implicated in autoimmune diseases (ADs) pathogenesis. However, its role in primary Sjögren's syndrome (pSS) remains unclear. This study aims to identify pyroptosis-related gene (PRG) signatures in pSS.

**Methods:**

Three datasets were obtained from the Gene Expression Omnibus (GEO) database to analyze the gene expression profiles in pSS. Differentially expressed genes (DEGs) were intersected with PRGs to identify pyroptosis-related DEGs (PRDEGs). Functional enrichment was assessed using Gene Ontology (GO) and Kyoto Encyclopedia of Genes and Genomes (KEGG). Key genes were identified using the least absolute shrinkage and selection operator (LASSO) and support vector machine (SVM) analyses. A diagnostic model was constructed using logistic regression. mRNA–microRNA (miRNA) and mRNA–transcription factor (TF) interaction networks were constructed. Immune cell infiltration (ICI) was analyzed using cell-type identification by estimating relative subsets of RNA transcripts (CIBERSORTs) and single-sample gene set enrichment analysis (ssGSEA). Experimental validation was performed in nonobese diabetic (NOD)/ShiLtj mice using reverse transcription quantitative polymerase chain reaction (RT-qPCR), western blotting, and immunofluorescence and was further validated using immunofluorescence in pSS patient samples.

**Results:**

A total of 489 DEGs were identified, of which 22 were pyroptosis-related. GO/KEGG analysis revealed enrichment in immune response regulation, pyroptosis, and the positive regulation of receptor signaling pathways. LASSO and SVM analyses identified eight key genes (*CRTAC1*, platelet-endothelial cell adhesion molecule-1 [*PECAM1*], *IRF2*, *GZMA*, *IFI16*, absent in melanoma 2 [*AIM2*], *TNF*, and macrophage-expressed gene 1 [*MPEG1*]), which were incorporated into a diagnostic model that demonstrated strong discriminatory performance in both combined and validation datasets. Experimental validation confirmed significant increased expressions of *PECAM1*, *IFI16*, *AIM2*, and *MPEG1* in the salivary glands from both NOD mice and pSS patients.

**Conclusions:**

PECAM1, IFI16, AIM2, and MPEG1 were identified as PRG signatures and potential biomarkers in pSS, providing novel insights into pSS pathogenesis.

## 1. Introduction

Primary Sjögren's syndrome (pSS) is a chronic and systemic autoimmune disease (AD) characterized by decreased secretion from lacrimal and salivary glands. The female-to-male ratio is approximately 14:1, with an estimated prevalence of 0.01%−0.05% [1]. The primary clinical manifestations are xerophthalmia and xerostomia. Moreover, many patients exhibit systemic symptoms, such as fatigue and multiorgan damage beyond the exocrine glands, affecting the skin, lungs, kidneys, gastrointestinal tract, and nerves [2], severely impairing their quality of life. Despite extensive research, the precise pathogenesis of pSS remains poorly understood.

Pyroptosis is a form of programmed cell death driven by inflammasome activation and characterized by cell swelling, membrane rupture, and the release of intracellular contents and inflammatory cytokines such as interleukin (IL)-1β and IL-18 [3]. It has been implicated in various inflammatory and ADs [4, 5]. Previous studies have reported increased expression of nucleotide-binding oligomerization domain-like receptor family pyrin domain-containing 3 (NLRP3) inflammasome components in pSS, suggesting a potential link between pyroptosis and disease pathogenesis [6]. However, the specific gene signatures and regulatory mechanisms underlying pyroptosis in pSS remain poorly defined.

In this study, we aim to identify pyroptosis-related gene (PRG) signatures in pSS through bioinformatic analysis and experimental validation, laying a foundation for future studies exploring their roles in disease mechanisms and as potential therapeutic targets.

## 2. Materials and Methods

### 2.1. Data Acquisition

**Figure 1:**
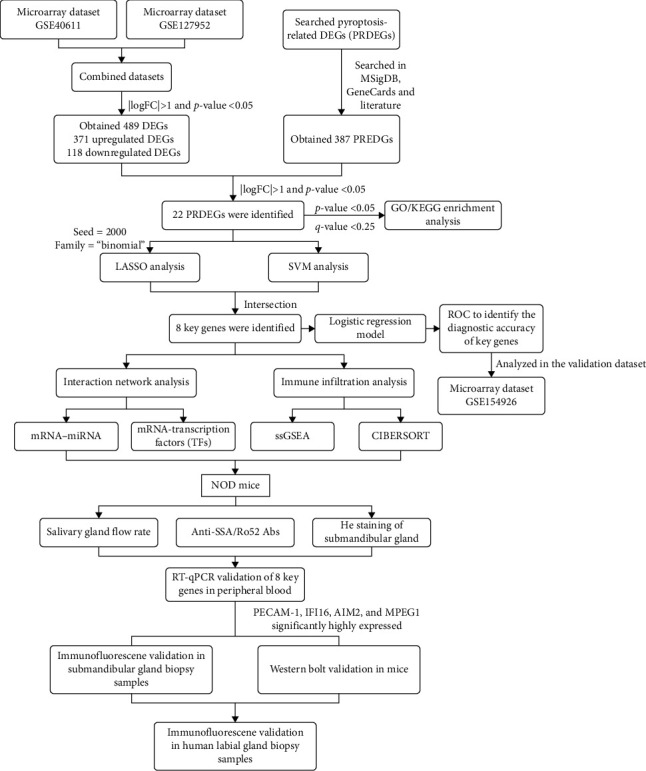
Schematic representation of the study workflow. Datasets GSE40611 and GSE127952 were downloaded from GEO and integrated for analysis. DEGs were identified and intersected with PRGs to obtain PRDEGs. The main enriched pathways were analyzed by GO and KEGG enrichment analyses. Key genes were identified through the intersection of LASSO and SVM analyses. A logistic regression model was constructed to assess the predictive value of key genes. mRNA–miRNA and mRNA–TF interaction networks were established. The ICI profile was analyzed using CIBERSORT and ssGSEA. The expression of key genes was quantified in NOD mice by RT-qPCR and further validated in submandibular glands via immunofluorescence and western blot analyses. Labial gland biopsy samples from patients with pSS were also used for validation through immunofluorescence. DEGs, differentially expressed genes; GO, Gene Ontology; ICI, immune cells infiltration; KEGG, Kyoto Encyclopedia of Genes and Genomes; LASSO, least absolute shrinkage and selection operator; PRDEGs, pyroptosis-related differentially expressed genes; PRGs, pyroptosis-related genes; SVM, support vector machine; TF, transcription factors.

The workflow of this study is illustrated in Figure 1. Three datasets related to pSS (GSE40611 [7], GSE127952, and GSE154926 [8]) were obtained from the Gene Expression Omnibus (GEO) database (https://www.ncbi.nlm.nih.gov/geo/; Table 1). GSE40611 contained 35 parotid tissue samples, including 17 from pSS patients and 18 from healthy controls (HCs). GSE127952 included 14 minor salivary gland samples, consisting of eight from pSS patients and six from HCs. GSE154926 contained 50 minor salivary gland samples, comprising 43 from pSS patients and seven from HCs. Datasets GSE40611 and GSE127952 were merged to create the combined dataset, whereas GSE154926 was designated as the validation dataset.

Twenty-seven PRGs were identified using the term “pyroptosis” in the Molecular Signatures Database (MSigDB). The GeneCards database was queried using the term “pyroptosis,” resulting in the identification of 380 PRGs. An additional 31 PRGs were obtained from Wu et al. [9]. By merging the PRGs from these three sources, a total of 387 PRGs were obtained for subsequent analyses (Supporting Information 1: Table S1). The complete list of PRGs with corresponding data sources is provided in Supporting Information 2: Table S1a (Excel file).

### 2.2. Identification of Differentially Expressed Genes (DEGs) Related to Pyroptosis

Initially, the R package “sva” was employed to eliminate batch effects between datasets GSE40611 and GSE127952. Subsequently, the “FactoMineR” and “factoextra” R packages were applied to analyze the datasets before and after batch effect correction. Differential expression analysis of the combined dataset was performed using the R package “limma.” DEGs were screened using the criteria of *p*  < 0.05 and |logFC| > 1.

All DEGs were intersected with PRGs to identify pyroptosis-related DEGs (PRDEGs) and the results were visualized using a Venn diagram. A volcano plot was generated using the R package “ggplot2” to visualize the results of the differential expression analysis and heatmaps were produced with the R package “pheatmap.”

### 2.3. Gene Ontology (GO) and Kyoto Encyclopedia of Genes and Genomes (KEGG) Analyses

GO and KEGG pathway enrichment analyses were conducted on the DEGs using the R package “clusterProfiler.” Statistical significance was defined as *p*  < 0.05 and false discovery rate (FDR, *q*-value) <0.05, with *p*-values adjusted using the Benjamini–Hochberg method.

### 2.4. Identification of Key Genes by Least Absolute Shrinkage and Selection Operator (LASSO) and Support Vector Machine (SVM) Analyses

To identify key PRGs among the PRDEGs, two machine learning algorithms were applied. LASSO [10] regression was performed using the R package “glmnet” [11]. Additionally, a SVM model was constructed [12]. The optimal feature set was selected based on the minimum classification error and maximum model accuracy. Genes identified by both LASSO and SVM-RFE were defined as key genes for downstream model development.

### 2.5. Construction and Evaluation of a Diagnostic Model

Logistic regression analysis was performed on the key genes to evaluate their association with pSS diagnosis. A multivariable logistic regression model was then constructed based on the selected genes. A nomogram was constructed using the R package “rms” to provide a visual and clinically interpretable diagnostic tool. To assess the performance and clinical utility of the model, calibration curves and decision curve analysis (DCA) plots were generated by the R package “ggDCA.” The diagnostic accuracy of each gene was further evaluated using receiver operating characteristic (ROC) curves and the corresponding area under the curve (AUC) values in both combined and validation datasets.

### 2.6. Establishment of mRNA–MicroRNA (miRNA) and mRNA–Transcription Factor (TF) Interaction Networks

The interactions between miRNAs and key genes were predicted using the ENCORI database (https://rnasysu.com/encori/). Only mRNA–miRNA interactions supported by at least three independent platforms were retained. A corresponding interaction network was then constructed.

TFs targeting key genes were identified using the ChIPBase (https://rna.sysu.edu.cn/chipbase/) and hTFtarget databases (http://bioinfo.life.hust.edu.cn/hTFtarget). The resulting mRNA–TF interaction network was visualized using Cytoscape software.

### 2.7. Immune Infiltration Analysis (Cell-Type Identification by Estimating Relative Subsets of RNA Transcript [CIBERSORT] and Single-Sample Gene Set Enrichment Analysis [ssGSEA])

The gene expression matrix of the combined dataset was submitted to the CIBERSORT online platform (https://cibersortx.stanford.edu/) and analyzed using the LM22 signature gene matrix. Samples with a *p*-value < 0.05 were retained to generate the immune cell infiltration (ICI) matrix. Next, immune cell types with relative fractions greater than zero were retained to produce the final ICI matrix. Differences in the infiltration levels of the 22 immune cell types between the high- and low-risk groups (High group and Low group, respectively, defined based on the logistic regression model) were visualized using box plots, while correlations between immune cells and key genes were represented by correlation heatmaps.

GSEA was further extended to ssGSEA, which calculated enrichment scores to quantify the degree of gene set activation in individual samples. The relative abundances of 28 immune cell types were quantified in the combined dataset using the ssGSEA algorithm. Relationships between the key gene expression levels and ssGSEA-derived immune cell scores were analyzed.

### 2.8. Experimental Animals

Female BALB/c mice (specific pathogen-free [SPF] grade), aged 6–8 weeks, were supplied by the Experimental Animal Center of Fujian University of Traditional Chinese Medicine. Female nonobese diabetic (NOD)/ShiLtj mice (SPF-grade), aged 7 weeks, were purchased from Jiangsu Jicui Yaokang Biotechnology Co., Ltd. (Approval Number: SCXK [Su] 2023-0009) and housed in the SPF-grade barrier facility at the Animal Experiment Center of Fujian University of Traditional Chinese Medicine. The housing conditions were maintained at a temperature of 24 ± 2°C, with a 12-h light/dark cycle and humidity levels of 45%–55%. Animals had unrestricted access to standard feed and water. The animals were randomly assigned to experimental and control groups using a computer-generated randomization protocol. All animals were housed under identical environmental conditions, including temperature, humidity, and light/dark cycles, to ensure uniformity across groups. Researchers performing the experiments were blinded to the group assignments to minimize potential bias.

### 2.9. Human Samples

Lacrimal gland biopsy samples were obtained from three pSS patients and three healthy donors at Fuzhou University Affiliated Provincial Hospital. All patients fulfilled the 2016 ACR/EULAR classification criteria for pSS [13]. Human samples were randomly selected from eligible participants and matched for age and sex to minimize confounding. The mean age of the pSS group was 48 ± 3.5 years, while that of the control group was 45 ± 2.7 years. All participants were female. Sampling procedures were standardized, ensuring that biopsy collection, sample preservation, and downstream analyses were conducted under identical conditions. Researchers analyzing the samples were blinded to their group assignments to ensure objective data interpretation.

### 2.10. Measurement of Salivary Gland Flow Rate in NOD/ShiLtj Mice

The mice were anesthetized with isoflurane prior to the measurement. Subsequently, the mice received an intraperitoneal injection of sterile pilocarpine at a dosage of 5 mg/kg body weight. Five minutes later, sterile cotton balls preweighed at 10 mg each were placed into the buccal cavity of the mice and saliva was collected over a 5 min period. The salivary flow rate was measured using the wet–dry weight method. The initial weight of the dry cotton balls (*W*_1_) and the weight after saliva collection (*W*_2_) were used to calculate the saliva volume as the weight difference (*ΔW* = *W*_2_ − *W*_1_), which represented the amount of saliva secreted over 5 min.

### 2.11. ELISA

The mice were anesthetized with isoflurane, and blood samples were collected and centrifuged to isolate serum. Serum samples, reference standards, and sample diluents were added to a polystyrene plate. Freshly diluted enzyme-labeled antibodies were added to each reaction well. The plate was incubated at 37°C for 1 h, and then, washed five times. Substrate Solutions A and B were added to each well to initiate color development. The plate was incubated in the dark at 37°C for 15 min. The reaction was terminated by adding 50 μL of stop solution into each well. The optical density (OD) at 450 nm was measured using a microplate reader, with blank control wells used for background correction. The concentration of anti-SSA/Ro antibodies in each sample was calculated.

### 2.12. Hematoxylin and Eosin (HE) Staining

After anesthesia and the blood collection, the mice were euthanized via cervical dislocation. The submandibular glands were excised, dehydrated using graded ethanol, embedded in paraffin, and sectioned into 4 μm-thick sections. The sections were deparaffinized in xylene, rehydrated through graded ethanol, and stained with HE. Neutral balsam was applied for mounting, and lymphocytic infiltration in the submandibular gland tissue was examined under a light microscope.

### 2.13. Reverse Transcription Quantitative Polymerase Chain Reaction (RT-qPCR)

Total RNA was extracted from peripheral blood using the Trizol reagent (Vazyme, China) and subsequently reverse-transcribed into cDNA. PCR amplification was performed using gene-specific primers (Supporting Information 1: Table S2). RNA concentration and purity were assessed using a NanoDrop 2000 spectrophotometer (Thermo Fisher Scientific, USA), and only samples with an A260/A280 ratio between 1.8 and 2.0 were used for downstream analysis. RT was performed using a commercial reverse transcription kit (Thermo Fisher Sientific, USA) and quantitative PCR amplification was conducted using SYBR Green chemistry on a QuantStudio 5 real-time PCR system. The specificity of the PCR products was confirmed by melt curve analysis. Each reaction was performed in triplicate, and negative controls were included in parallel. Gene expression levels were normalized to GAPDH and relative expression was calculated using the 2^−*ΔΔ*Ct^ method.

### 2.14. Western Blot

Total protein was extracted from mouse submandibular glands using RIPA lysis buffer supplemented with protease and phosphatase inhibitors. Protein concentration was determined using a BCA Protein Assay Kit (Beyotime, China, P0011). Equal amounts of protein samples were separated by 10% SDS-PAGE and transferred onto polyvinylidene difluoride membranes. The membranes were blocked at room temperature for 30 min using a protein-free blocking buffer (Yeasen Biotechnology, China, PS108P).

The membranes were incubated overnight at 4°C with primary antibodies, including anti-NLRP3 (Abcam, USA, ab263899), anti-IFI16 (Invitrogen, USA, PA5-89569), anti-macrophage-expressed gene 1 (MPEG1; MyBioSource, USA, MBS9410829), anti-absent in melanoma 2 (AIM2; MyBioSource, USA, MBS177917), anti-CD31/platelet-endothelial cell adhesion molecule-1 (PECAM-1; Novus Biologicals, USA, NB100-2284), and anti-β-actin (RunBolead, China, A0101). After washing, HRP-conjugated secondary antibodies (goat anti-rabbit IgG-HRP, RunBolead, China, S0101 and goat anti-mouse IgG-HRP, RunBolead, China, S0100) were applied. Target proteins were visualized using an enhanced chemiluminescence (ECL) detection kit (Meilunbio, China, MA0187).

### 2.15. Immunofluorescence Analysis

Tissue samples from mice and clinical sources were fixed in 4% paraformaldehyde, dehydrated, embedded in paraffin, and sectioned at a thickness of 4 μm. Sections were deparaffinized at room temperature, rehydrated, subjected to heat-induced antigen retrieval, permeabilized with 0.5% Triton X-100, and blocked with goat serum. Sections were incubated overnight at 4°C with primary antibodies, including anti-CD31/PECAM-1 (1:100, Novus Biologicals, USA), anti-AIM2 (1:500, MyBioSource, USA), anti-IFI16 (1:200, Invitrogen, USA), and anti-MPEG1 (1:200), MyBioSource, USA). On the second day, fluorescently labeled secondary antibodies were added and incubated in the dark, followed by DAPI staining to visualize the nuclei. The sections were mounted and examined under an inverted fluorescence microscope. For each sample, five random fields were selected for imaging with a fluorescence inverted microscope under identical exposure and gain settings. The images were analyzed using ImageJ software. Mean fluorescence intensity was calculated as the integrated density (IntDen) divided by the area of the region of interest (Mean = IntDen/area). This quantification approach was applied consistently across all samples.

### 2.16. Statistical Methods

All data were processed and analyzed using R software (version 4.2.2). The independent Student's *t*-test was applied to compare normally distributed continuous variables, whereas the Mann–Whitney *U* test was used for nonnormally distributed data. The Kruskal–Wallis test was employed to compare differences across three or more groups. Categorical variables between two groups were compared using the chi-square test or Fisher's exact test. Spearman correlation analysis was conducted to evaluate correlations between variables. Unless otherwise specified, all tests were two-tailed and a *p*-value < 0.05 was considered statistically significant.

## 3. Results

### 3.1. Datasets Merging and Correction

Batch effects in the integrated datasets GSE40611 and GSE127952 were corrected using the R package “sva.” Boxplots and principal component analysis (PCA) were used to visualize and compare the datasets before and after batch adjustment (Supporting Information 1: Figure S1). The results demonstrated that batch effects were effectively removed, indicating successful datasets integration.

### 3.2. Identification of DEGs Associated With Pyroptosis in pSS

**Figure 2:**
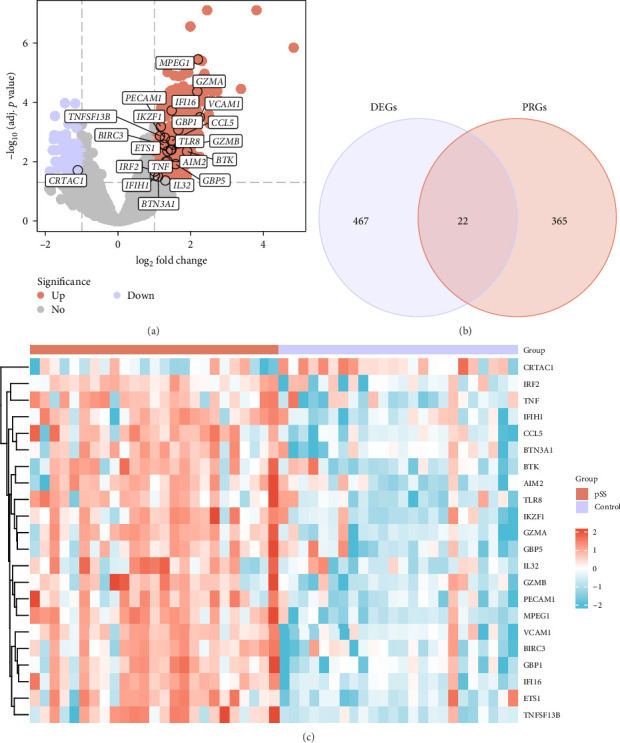
Analysis of pyroptosis-related differentially expressed genes. (a) Volcano plot of DEGs between the pSS and control groups from the combined dataset. Labeled genes represent the PRDEGs identified in the analysis. (b) Venn diagram showing the overlap between DEGs and PRGs. (c) Heatmap depicting the expression patterns of PRDEGs in the pSS and control groups. DEGs, differentially expressed genes; PRDEGs, pyroptosis-related differentially expressed genes; PRGs, pyroptosis-related genes.

Differential expression analysis between the pSS and control groups was performed on the combined dataset using the R package “limma.” A total of 489 DEGs were identified, including 371 upregulated and 118 downregulated genes. A volcano plot was generated to visualize the results (Figure 2a).

PRGs were obtained using a multisource approach to ensure their completeness and relevance: (1) MSigDB: genes explicitly associated with pyroptosis were extracted from the MSigDB database. (2) GeneCards: the keyword “pyroptosis” was used to identify additional related genes. (3) Literature: PRGs were obtained manually from Wu et al. [9]. Through this integrative approach, 387 PRGs were identified (Supporting Information 1: Table S1), encompassing key regulators of pyroptosis, such as caspases (*CASP1*, *CASP3*, *CASP4*, *CASP5*, and *CASP8*), inflammasome components (*NLRP1*, *NLRP3*, *AIM2*, and *IFI16*), pro-inflammatory cytokines (*IL-1B*, *IL-18*, and *TNF*), and regulatory molecules (*GSDMD*, *PYCARD*, *IRF2*, and *ETS1*).

Twenty-two PRDEGs were identified by intersecting the DEGs with PRGs (Figure 2b), including *CCL5*, *ETS1*, *IFIH1*, *CRTAC1*, *PECAM1*, *IKZF1*, *GBP1*, *IRF2*, *IL32*, *VCAM1*, *GZMA*, *BTK*, *IFI16*, *AIM2*, *TNF*, *BTN3A1*, *GZMB*, *BIRC3*, *TLR8*, *GSF13B*, *MPEG1*, and *GBP5*. The expression patterns of PRDEGs in pSS and control samples were compared in the combined dataset and visualized in a heatmap (Figure 2c).

### 3.3. GO and KEGG Enrichment Analysis of PRDEGs

**Figure 3:**
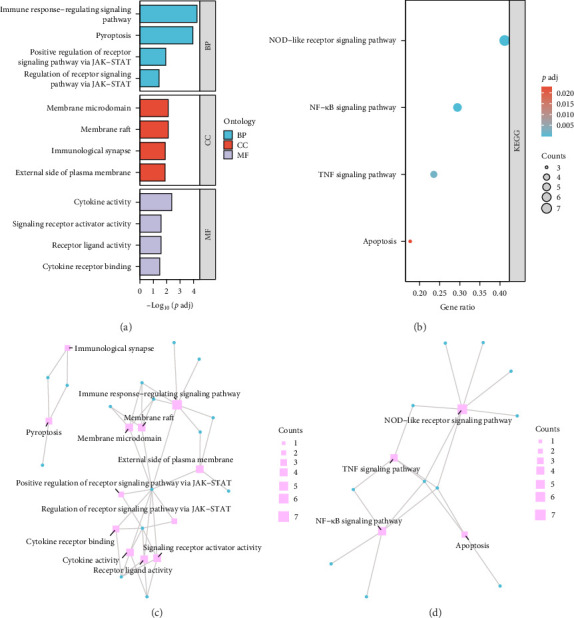
GO and KEGG enrichment analysis of PRDEGs. (a) GO enrichment analysis of PRDEGs. GO terms are classified into three categories: BP, CC and MF. The bar chart displays the top significantly enriched GO items, ranked by adjusted *p*-values. (b) KEGG pathway enrichment analysis for PRDEGs. The bubble plot represents significantly enriched pathways, with bubble size corresponding to the number of genes involved in each pathway. The bubble color gradient represents statistical significance, with bluer hues indicating higher enrichment significance. (c) Network diagram illustrating the GO enrichment analysis results for PRDEGs. Blue nodes represent specific PRDEGs, while red nodes indicate GO terms. Node size reflects the number of genes associated with each GO term. Edges illustrate interactions between genes and GO terms. (d) Network diagram depicting the KEGG enrichment analysis results for PRDEGs. Blue nodes represent PRDEGs, while red nodes denote KEGG pathways. Node size and edges represent the number of genes involvement and interconnections among pathways. GO, Gene Ontology; KEGG, Kyoto Encyclopedia of Genes and Genomes; PRDEGs, pyroptosis-related differentially expressed genes.

To explore the biological significance of 22 PRDEGs, GO and KEGG enrichment analyses were performed (Figure 3). GO enrichment analysis classified the PRDEGs under three functional domains: biological process (BP), molecular function (MF), and cellular component (CC; Table 2). The most significantly enriched BP terms included immune response-regulating signaling pathways, pyroptosis and the positive regulation of receptor signaling via the JAK-STAT pathway, highlighting the critical role of PRDEGs in immune modulation. The CC terms were predominantly associated with membrane microdomains, membrane rafts, immunological synapses, and the external side of the plasma membrane, suggesting that these genes may influence cell-cell communication and immune cell activation. Within the MF category, PRDEGs were significantly enriched in cytokine activity, receptor ligand activity, and cytokine receptor binding, which are key processes in inflammatory signaling (Figure 3a).

KEGG pathway enrichment analysis further revealed that PRDEGs are predominantly enriched in inflammatory and immune-related pathways (Table 2). The top enriched pathways included the NOD-like receptor (NLR) signaling pathway, NF-κB signaling pathway, and TNF signaling pathway (Figure 3b), suggesting that PRGs contribute to inflammatory and immune responses in pSS. The NLR signaling pathway plays a central role in inflammasome activation and pyroptotic cell death [14], whereas the NF-κB signaling pathway is a well-established mediator of chronic inflammation in ADs [15].

To visualize these relationships, GO and KEGG networks were constructed (Figure 3c,d). These networks provide an intuitive visualization of PRDEG enrichment patterns. Larger nodes represent GO or KEGG terms associated with a greater number of genes, while edges illustrating functional links between terms. The GO network (Figure 3c) highlights PRDEG involvement in immune system activation, while the KEGG network (Figure 3d) underscores their roles in inflammatory and immune responses. Taken together, these analyses elucidate the functional roles of PRDEGs and their involvement in pSS pathogenesis.

### 3.4. LASSO Model and SVM Analysis

**Figure 4:**
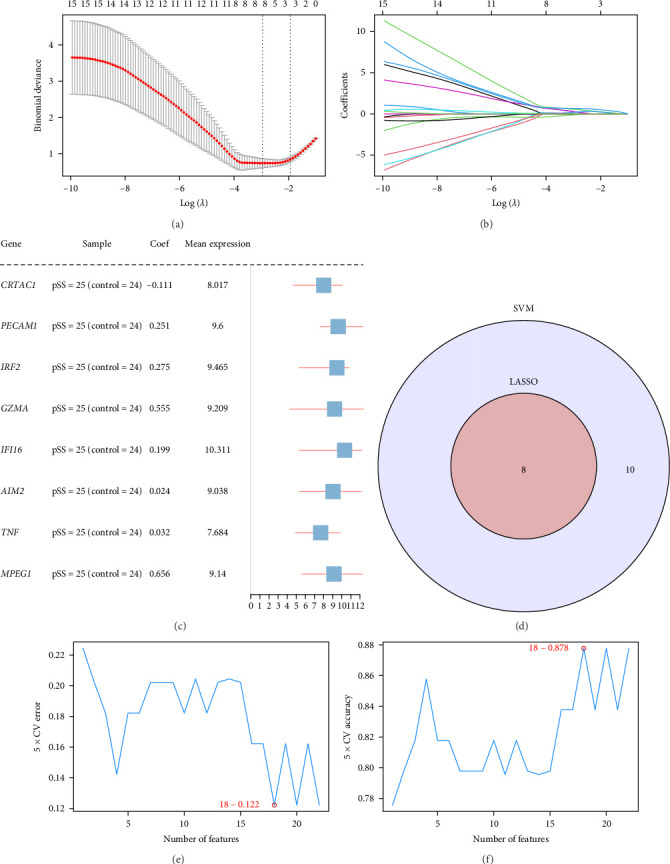
LASSO regression and SVM analysis of key genes. (a) LASSO regression analysis with tenfold cross-validation for identifying the optimal *λ* value in the prognostic model. (b) Coefficient profiles of the diagnostic model based on LASSO regression, showing how gene coefficients shrink with increasing penalty (log *λ*). (c) Forest plot displaying the coefficients and mean expression levels of selected genes in the LASSO–based diagnostic model. (d) Venn diagram showing the overlap between key genes identified by the SVM and LASSO algorithms. (e) Fivefold cross-validation curve showing the relationship between the number of selected genes and classification error rate in the SVM model. The model achieved its lowest error when 18 genes were included. (f) Fivefold cross-validation curve showing the relationship between the number of selected genes and model accuracy. The highest accuracy was reached when 18 genes were selected. LASSO, least absolute shrinkage and selection operator; SVM, support vector machine.

LASSO regression was employed to identify key genes (Figure 4a,b). The LASSO regression with tenfold cross-validation identified the optimal *λ* value for building a diagnostic model. At this threshold, eight genes with nonzero coefficients were selected (Figure 4a). The coefficient trajectories of candidate genes revealed their variation with increasing regularization strength (Figure 4b). The final diagnostic model consisted of eight key genes (*CRTAC1*, *PECAM1*, *IRF2*, *GZMA*, *IFI16*, *AIM2*, *TNF*, and *MPEG1*), whose coefficients and average expression levels were shown in the forest plot (Figure 4c).

An SVM model was constructed to determine the optimal gene set with the lowest classification error and highest accuracy (Figure 4e,f). The SVM model exhibited optimal accuracy when incorporating 18 genes, including *ETS1*, *CCL5*, *GZMB*, *IKZF1*, *IL32*, *VCAM1*, *BIRC3*, *GBP1*, *GBP5*, *BTK*, *CRTAC1*, *PECAM1*, *IRF2*, *GZMA*, *IFI16*, *AIM2*, *TNF*, and *MPEG1*. The overlap between the LASSO and SVM results yielded eight shared genes: CRTAC1, PECAM1, IRF2, GZMA, IFI16, AIM2, TNF, and MPEG1 (Figure 4d), which were, therefore, defined as key pyroptosis-associated genes.

### 3.5. Establishment of the Logistic Model and Diagnostic Value of Key Genes

**Figure 5:**
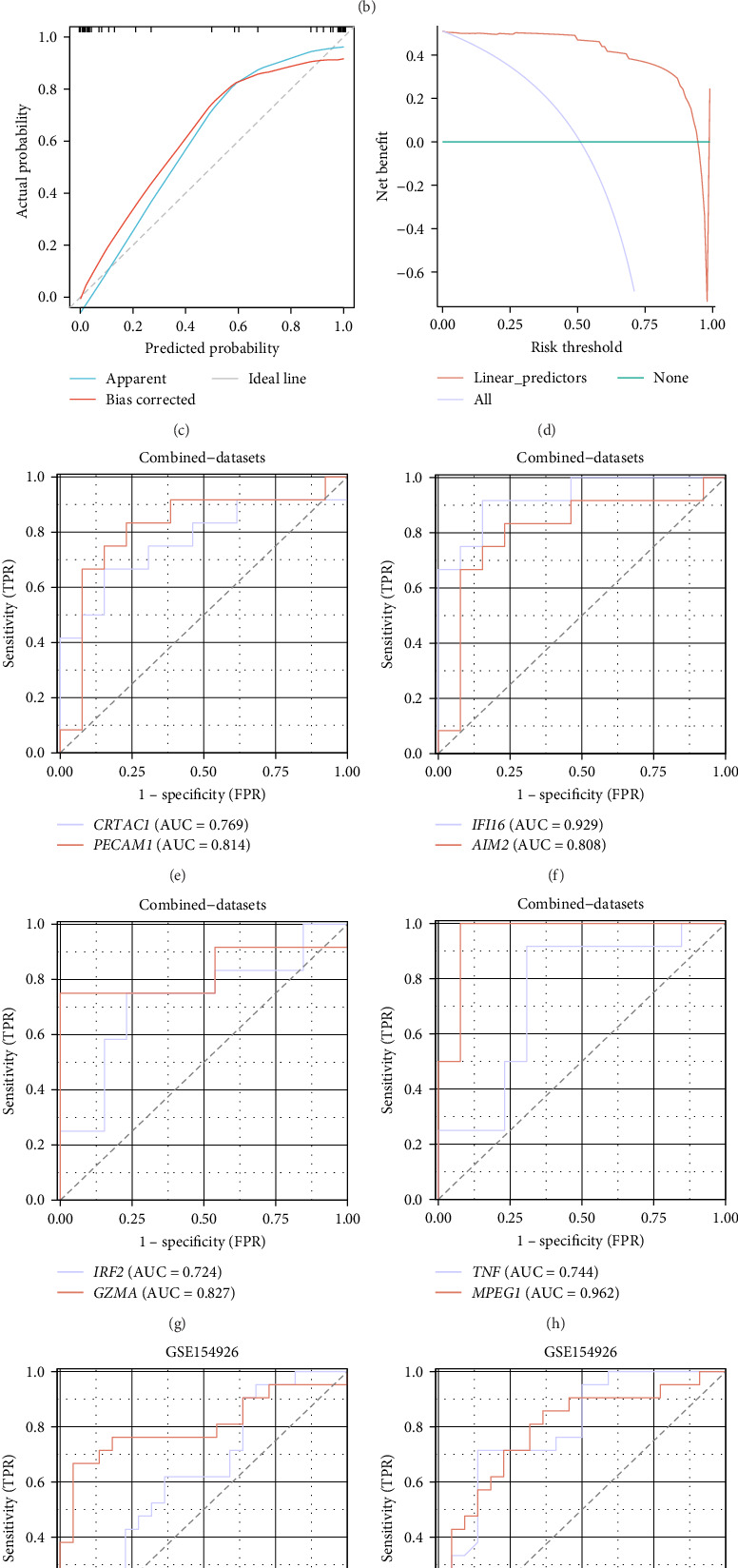
Construction and validation of a diagnostic model based on eight key pyroptosis-related genes. (a) Nomogram constructed using eight selected genes to predict the risk of pSS. Each gene corresponds to a specific point score and total points are used to estimate individual risk. (b) Forest plot showing the odds ratios (ORs), 95% confidence intervals (CIs), and *p*-values from univariate logistic regression analysis of the eight key genes in relation to pSS diagnosis. (c) Calibration curve assessing the consistency between predicted probabilities and actual observations. (d) DCA evaluating the clinical utility of the diagnostic model across a range of risk thresholds. (e–h) ROC curves of individual genes in the combined training datasets, with AUC values indicating diagnostic accuracy. The following genes were analyzed: *CRTAC1* and *PECAM1* (e); *IFI16* and *AIM2* (f); *IRF2* and *GZMA* (g); *TNF* and *MPEG1* (h). (i–l) ROC curves of the same genes in the validation dataset (GSE154926), confirming their diagnostic value. The following genes were analyzed: *CRTAC1* and *PECAM1* (i); *IFI16* and *AIM2* (j); *IRF2* and *GZMA* (k); *MPEG1* (l). AUC values closer to 1 indicate a stronger diagnostic performance. AUC thresholds were defined as follows: 0.5–0.7 indicates low diagnostic accuracy, 0.7–0.9 indicates moderate diagnostic accuracy, and >0.9 indicates high diagnostic accuracy. DCA, decision curve analysis; ROC, receiver operating characteristic curve.

To further evaluate the diagnostic potential of the eight key genes identified by LASSO and SVM, a diagnostic nomogram was constructed. As shown in Figure 5a, the nomogram integrates all eight genes (*CRTAC1*, *PECAM1*, *IRF2*, *GZMA*, *IFI16*, *AIM2*, *TNF*, and *MPEG1*) to generate an individual risk score. This score corresponds to the predicted probability of pSS. Univariate logistic regression analysis revealed that all eight genes were significantly associated with disease status (Figure 5b), with odds ratios ranging from 0.35 to 12.83, suggesting strong discriminative power.

To assess the reliability of the model, a calibration curve was plotted, demonstrating strong concordance between predicted probabilities and observed outcomes (Figure 5c). Furthermore, DCA demonstrated that the diagnostic model provides substantial net clinical benefit across a wide range of risk thresholds, supporting its clinical applicability (Figure 5d).

The diagnostic performance of each gene was further evaluated using ROC curves. In the combined dataset, several genes exhibited high diagnostic performance, with AUC values greater than 0.80, including IFI16 (AUC = 0.929), MPEG1 (AUC = 0.962), and PECAM1 (AUC = 0.814; Figure 5). To validate these findings, the diagnostic model was tested in the external validation dataset (GSE154926). The external validation confirmed the high diagnostic value of these genes, with consistent AUC values above 0.80 for most genes (Figure 5).

Together, these results indicate that the eight-gene diagnostic model is both accurate and generalizable and may serve as a promising tool for the early diagnosis of pSS.

### 3.6. Interaction Network of mRNA–miRNA and mRNA–TF

**Figure 6:**
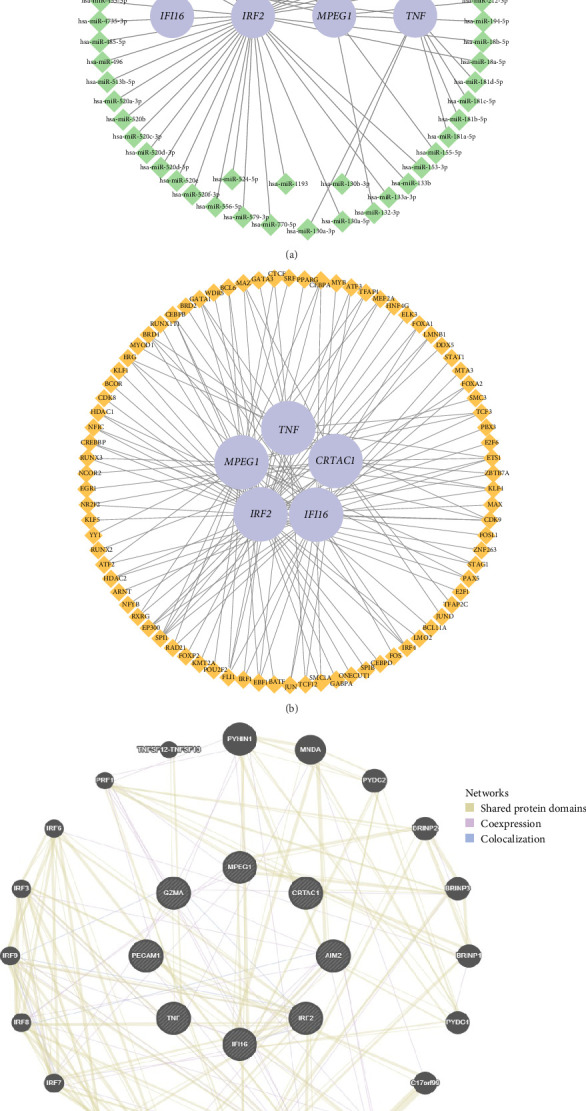
Interaction networks of mRNA–miRNA and mRNA–TF. (a) The mRNA–miRNA interaction network illustrates regulatory relationships between key genes and their associated miRNAs. Purple circles represent mRNA molecules, while green rectangles indicate miRNAs predicted to interact with them, supported by evidence from at least three independent platforms. (b) The mRNA–TF interaction network depicts transcriptional regulation of key genes. Purple circles represent mRNA molecules, while orange rectangles indicate TFs predicted to regulate them. (c) A functional association network generated by GeneMANIA platform (http://genemania.org), illustrating relationships between key genes and other genes with similar functions. The inner circle contains eight key genes, while the outer circle displays functionally related genes identified through coexpression, protein–protein interactions, and pathway analyses. Edges colors represent different types of predicted associations. TF, transcription factor.

To further investigate the upstream regulatory mechanisms of the eight key genes, mRNA–miRNA and mRNA–TF interaction networks were constructed (Figure 6). The mRNA–miRNA network illustrates potential regulatory relationships between the key genes and their targeting miRNAs. miRNAs interacting with the eight key genes were predicted based on established criteria, requiring evidence of interaction on at least three independent platforms to ensure reliability (Figure 6a). The resulting network comprised four mRNAs (*IFI16*, *IRF2*, *MPEG1*, and *TNF*) and 59 miRNAs. Detailed mRNA–miRNA interactions were provided in Supporting Information 1: Table S3. miRNAs typically function as posttranscriptional regulators by promoting mRNA degradation or inhibiting translation. Among these, IFI16 plays a crucial role in sensing cytoplasmic double-stranded DNA (dsDNA) and activating the inflammasome complex, ultimately leading to pyroptosis [16]. The presence of miRNAs targeting IFI16 suggests a possible mechanism for regulating its expression in pSS, potentially modulating the inflammatory response. Similarly, MPEG1, a known MPEG involved in immune defense [17], was also targeted by multiple miRNAs, indicating a potential regulatory role in immune activation and pyroptotic signaling.

The mRNA–TF interaction network (Figure 6b) provided insights into the transcriptional regulation of key genes. TFs bind to specific promoter or enhancer regions to modulate gene expression, thereby influencing inflammatory and pyroptotic pathways. In this research, TFs that bind to the key genes were identified by integrating data from CHIPBase and hTFtarget databases (Figure 6b). Only TFs with documented binding evidence were considered. The resulting interaction network included five mRNAs (*CRTAC1*, *IFI16*, *IRF2*, *MPEG1*, and *TNF*) and 82 TFs, with detailed mRNA–TF interactions presented in Supporting Information 1: Table S4. Notably, TFs such as STAT1 and IRF1 were found to be associated with key genes, suggesting their involvement in the immune and inflammatory regulation of pSS. For instance, STAT1, a key player in interferon signaling, was been reported to be upregulated in pSS and may contribute to the inflammatory state observed in the disease [18].

Furthermore, using the GeneMANIA prediction tool, we identified functionally similar genes based on shared coexpression, protein–protein interactions, and pathway associations with the key genes (Figure 6c). Together, these findings highlight the complex regulatory landscape governing PRGs in pSS. The identification of miRNAs and TFs potentially regulating key genes provides novel insights into both transcriptional and posttranscriptional control of pyroptosis.

### 3.7. ICI Analysis (CIBERSORT and ssGSEA)

**Figure 7:**
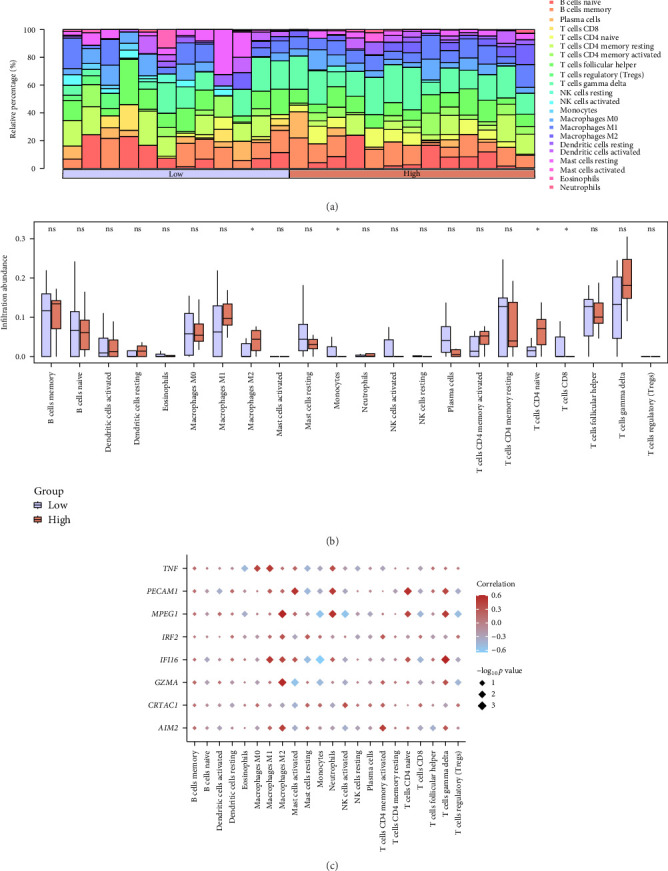
Immune cell infiltration analysis in the combined dataset using the CIBERSORT algorithm. (a) Stacked bar chart depicting immune cell composition in the High and Low groups within the combined dataset, as estimated using the CIBERSORT algorithm. (b) Box plot comparing the abundance of immune cell types between the High and Low groups. (c) Correlation heatmap showing the relationships between the expression levels of the eight key genes and the infiltration levels of 22 immune cell types. The color scale indicates the correlation coefficient and dot size reflects statistical significance. ns, not significant; *⁣*^*∗*^*p*  < 0.05.

To investigate the immune microenvironment associated with PRG expression, we performed ICI analysis using the CIBERSORT and ssGSEA algorithms. Based on the predicted probability from the logistic regression model, pSS samples from the combined dataset were stratified into high- and low-risk groups (High and Low group, respectively). As shown in Figure 7a, the relative proportions of 22 immune cell types varied across samples. Comparison between the High and Low groups revealed that M2 macrophages and naive CD4+ T cells were significantly more abundant in the High group (Figure 7b). To further explore gene-immune cell associations, a correlation heatmap was constructed, showing that multiple key genes were correlated with different immune cell subsets (Figure 7c). These findings suggest that PRGs may influence ICI and activation in the pSS microenvironment.

**Figure 8:**
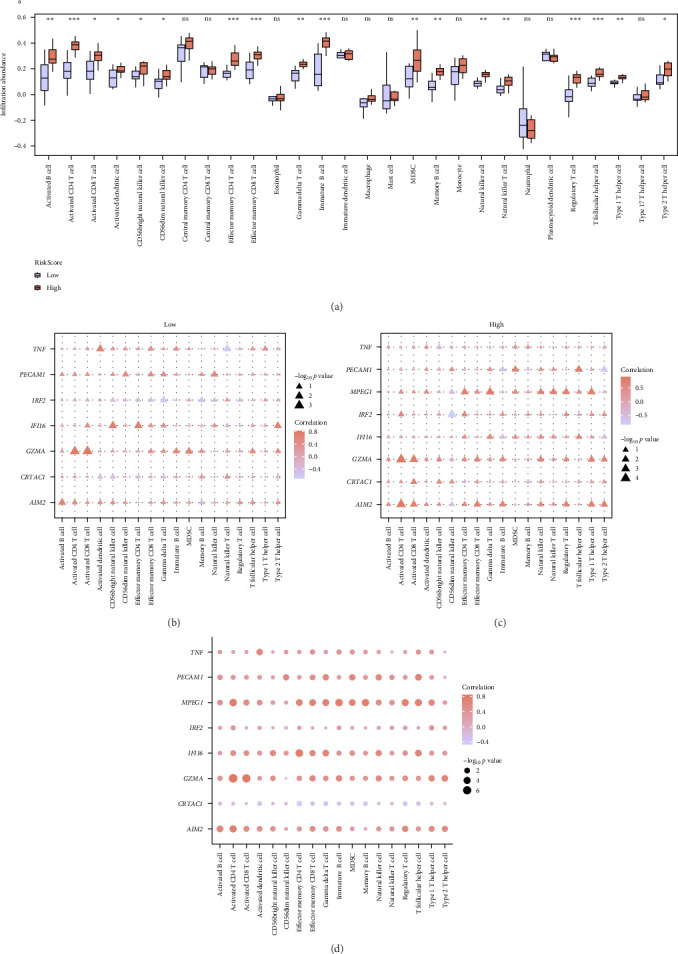
Immune cell infiltration analysis in the combined dataset using ssGSEA. (a) Boxplot showing the differences in ssGSEA-derived immune cell infiltration abundance between the High and Low groups. (b, c) Heatmap illustrating the correlation between key gene expression and immune cell signatures in the Low group (b) and High group (c), respectively. Triangle size indicates −log_10_*p* value; color scale represents correlation coefficient. (d) Heatmap summarizing the correlations between eight key genes and immune cell signatures across all samples. ns, no significance; *⁣*^*∗*^*p*  < 0.05, *⁣*^*∗∗*^*p*  < 0.01, *⁣*^*∗∗∗*^*p*  < 0.001.

As shown in Figure 8a, multiple immune cell types (activated B cells, activated CD4+ T cells, activated CD8+ T cells, myeloid-derived suppressor cells [MDSCs], and natural killer T cells) were significantly enriched in the High group compared to the Low group, suggesting a more activated and immunoregulatory microenvironment. Correlation analysis revealed that several key genes, including IFI16, PECAM1, and AIM2, were positively associated with activated immune cell subsets, particularly within the High group (Figure 8). These results indicate that PRDEGs may not only serve as diagnostic markers but also contribute to shaping the immune landscape in pSS.

### 3.8. Experimental Validation of PRGs in NOD/ShiLtj Mice and pSS Patients

**Figure 9:**
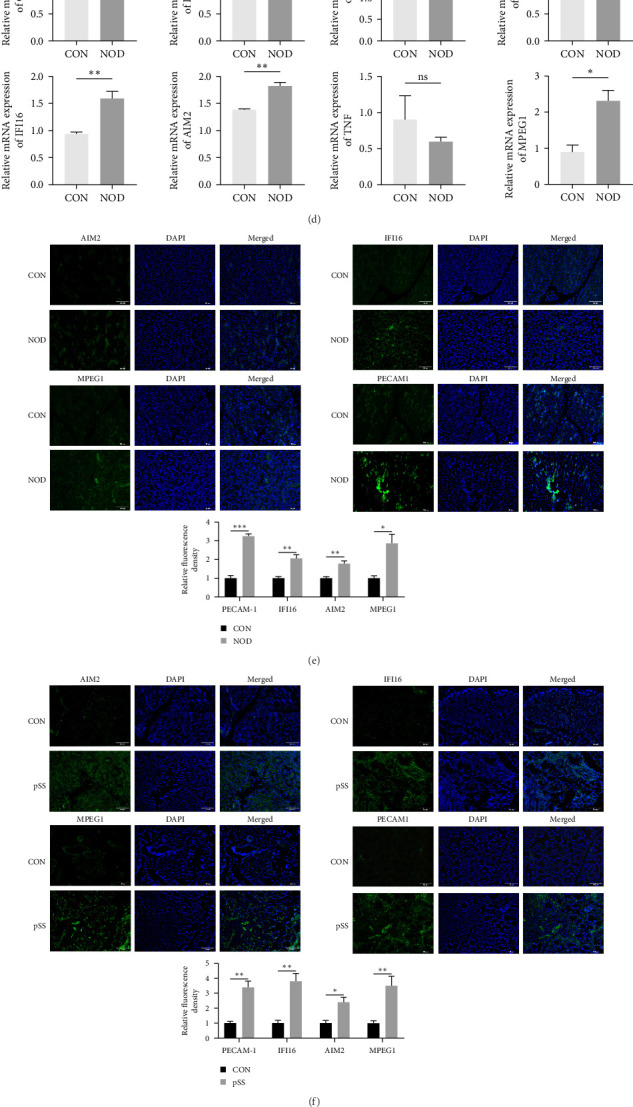
Experimental validation of pyroptosis-related genes in NOD/ShiLtj mice and pSS patients. (a) Salivary gland flow rate in different groups of mice (*n* = 3 per group). (b) Serum levels of anti-SSA/Ro antibodies measured by ELISA in different groups of mice (*n* = 3 per group). (c) Representative HE staining images of submandibular glands showing lymphocytic infiltration in NOD mice. (d) RT-qPCR analysis of PECAM1, IFI16, AIM2, and MPEG1 mRNA expression in mouse peripheral blood (*n* = 3 per group). (e) Immunofluorescence staining of PECAM1, IFI16, AIM2, and MPEG1 in mouse submandibular glands (*n* = 4 per group). The fluorescence intensity was measured and normalized against DAPI-stained nuclei. (f) Immunofluorescence staining of PECAM1, IFI16, AIM2, and MPEG1 in labial gland biopsy samples from pSS patients and healthy controls (*n* = 3 per group). The fluorescence intensity was measured and normalized against DAPI-stained nuclei. (g) Western blot analysis of NLRP3, PECAM1, IFI16, AIM2, and MPEG1 expression in mouse submandibular glands (*n* = 4 per group). CON, control group. *⁣*^*∗*^*p* < 0.05, *⁣*^*∗∗*^*p* < 0.01, and *⁣*^*∗∗∗*^*p* < 0.001.

To experimentally validate the findings from bioinformatic analyses, we conducted a series of validation experiments. First, the NOD/ShiLtj mouse model of pSS exhibited hallmark features of the disease, including significantly reduced salivary flow rates (Figure 9a), elevated serum anti-SSA/Ro antibody levels (Figure 9b), and histological evidence of lymphocytic infiltration and acinar destruction in the submandibular glands (Figure 9c).

RT-qPCR analysis revealed that the mRNA expression levels of PECAM1, IFI16, AIM2, and MPEG1 were significantly upregulated in peripheral blood from NOD mice compared to controls (Figure 9d). To further verify these findings at the protein level, western blotting was performed on submandibular gland tissues from NOD and control mice. Protein levels of PECAM1, IFI16, AIM2, and MPEG1 were significantly elevated in NOD mice, as confirmed by western blotting and quantitative analysis (Figure 9g). In addition, NLRP3, a key inflammasome component, were also upregulated in NOD mice (Figure 9g). Immunofluorescence staining of submandibular gland tissues further confirmed increased protein expression of PECAM1, IFI16, AIM2, and MPEG1 in NOD mice. Quantitative analysis demonstrated statistically significant differences (Figure 9e).

To confirm these results in human tissues, we analyzed labial gland biopsy samples from pSS patients and HCs. Immunofluorescence staining showed markedly increased expression of PECAM1, IFI16, AIM2, and MPEG1 in the pSS group, with quantitative results supporting these observations (Figure 9f). These findings aligned with results observed in mice and provide robust validation of PECAM1, IFI16, AIM2, and MPEG1 as pyroptosis-associated biomarkers in both experimental and clinical contexts of pSS.

## 4. Discussion

Our findings support the involvement of pyroptosis in pSS by identifying four key PRGs (*PECAM1*, *IFI16*, *AIM2*, and *MPEG1*) that were significantly upregulated in pSS patients and NOD mice. While bioinformatic analysis identified eight PRDEGs as key candidates, only four exhibited significant differential expression in experimental validation. This discrepancy may be attributed to several factors. (1) Posttranscriptional or posttranslational regulation. Although *CRTAC1*, *IRF2*, *GZMA*, and *TNF* were identified at the transcriptomic level, their expression may be modulated through posttranscriptional or translational mechanisms, which could explain the lack of detectable differences at the mRNA or protein levels. (2) Tissue-specific or conditional expression. Certain genes may exhibit differential expression only in specific cell populations or under specific inflammatory contexts not fully captured in our validation experiments. (3) Statistical thresholds used in bioinformatic analysis may not always reflect the functional relevance in disease pathology. (4) Potential functional roles beyond differential expression. These candidate genes may still contribute to disease progression through regulatory or signaling interactions with other PRGs, despite lacking strong differential expression.

Nevertheless, our findings validated that *PECAM1*, *IFI16*, *AIM2*, and *MPEG1* represent a PRG signature in pSS. PECAM1, also known as CD31, is a 130 kDa transmembrane protein expressed on the surface of diverse cell types, including granulocytes, monocytes, platelets, and endothelial cells [19]. It is involved in leukocyte migration, inflammation, coagulation, and platelet function [20]. Furthermore, PECAM1 has been implicated in the pathogenesis of ADs. A previous study demonstrated that in rheumatoid arthritis, lactate-activated PECAM1 signaling induced metabolic changes and triggered autophagy in endothelial cells [21].

IFI16 and AIM2 are both members of the AIM2 like receptor family. They function as intracellular DNA sensors, detecting pathogenic DNA within both the nucleus and cytoplasm. Upon recognition of dsDNA, they initiate inflammasome assembly by interacting with the adaptor protein ASC and procaspase-1. This leads to the cleavage of GSDMD and the induction of pyroptosis [22].

Previous studies have shown that IFI16 and AIM2 can bind to neutrophil extracellular traps (NETs), rendering them resistant to degradation and exacerbating autoimmune responses [23]. Additional studies have demonstrated that anti-IFI16 antibodies are highly specific to pSS and correlate with disease severity. Moreover, IFI16 expression is significantly elevated in the salivary glands of pSS patients compared to HCs [24]. Further studies have reported that AIM2 inflammasome, when activated by self-genomic DNA, can induce autoimmune responses in lacrimal gland myoepithelial cells and salivary gland epithelial cells in pSS [25, 26]. Collectively, these findings indicate that both IFI16 and AIM2 play potential roles in the pathogenesis of pSS, although their precise mechanisms remain to be fully elucidated.

MPEG1, also known as perforin-2, belongs to the membrane attack complexes/perforin (MACPF) superfamily. MPEG1 functions as a pore-forming protein involved in innate immunity and plays a critical role in host defense against invading viruses and bacteria [27]. Recent studies have demonstrated that MPEG1 is essential for antigen processing and cross-presentation in dendritic cells, a key process in adaptive immunity [28]. However, the role of MPEG1 in the pathogenesis of pSS remains unexplored.

Given their involvement in pyroptosis and immune regulation, these four key genes hold potential as clinical biomarkers and therapeutic targets for pSS. The elevated expression of IFI16 and AIM2 in pSS patients suggests their utility in disease diagnosis and severity assessment, particularly given the association of anti-IFI16 antibodies with disease progression. Additionally, targeting these pyroptosis-associated genes may provide novel therapeutic avenues for modulating immune responses in pSS, warranting further investigation into their functional roles in disease pathogenesis and treatment strategies. Future studies should focus on validating these targets in large patient cohorts and exploring small-molecule inhibitors or monoclonal antibodies as potential treatments.

Our study had several limitations. First, the relatively small sample sizes of the three datasets may have introduced bias in identifying key genes. Although we integrated two GEO datasets (GSE40611 and GSE127952) to enhance statistical power and applied batch effect correction, the limited sample size of the training cohort may still affect the robustness of feature selection and raise the risk of model overfitting. To mitigate this concern, we performed external validation using an independent dataset (GSE154926) and further validated the expression of key genes in both NOD/ShiLtj mice and human labial gland biopsy samples. These biologically independent systems support the diagnostic relevance of the identified genes. Nonetheless, future studies involving larger, multicenter patient cohorts will be essential to confirm the robustness and generalizability of the proposed PRG signature. Additionally, this study did not include mechanistic experiments such as gene knockdown, knockout, or overexpression. Future research should incorporate functional assays to further elucidate the regulatory mechanisms of pyroptosis in the context of pSS pathogenesis.

In conclusion, our study highlights four key genes related to pyroptosis in Sjögren's syndrome. These findings provide new insights into the molecular mechanisms and signaling pathways underlying pyroptosis in pSS. This work may contribute to a deeper understanding of pSS pathogenesis and support the development of novel targeted therapeutic strategies.

## Figures and Tables

**Table 1:** GEO dataset information list.

Category	GSE40611	GSE127952	GSE154926
Platform	GPL570	GPL20995	GPL16791
Species	*Homo sapiens*	*Homo sapiens*	*Homo sapiens*
Tissue	Parotid tissue	Minor salivary gland	Minor salivary glands
Samples in control group	18	6	7
Samples in pSS group	17	8	43
Reference	PMID: 23116360	—	—

**Table 2:** GO/KEGG enrichment analysis results.

Ontology	ID	Description	Gene ratio	Bg ratio	*p*-Value	*p*.adjust	*q*-Value
BP	GO:0002764	Immune response-regulating signaling pathway	7/22	482/18800	8.51E−07	5.43E−05	3E−05
BP	GO:0070269	Pyroptosis	3/22	22/18800	2.11E−06	0.00011	6.08E−05
BP	GO:0046427	Positive regulation of receptor signaling pathway via JAK-STAT	2/22	45/18800	0.001255	0.01191	0.006574
BP	GO:0046425	Regulation of receptor signaling pathway via JAK-STAT	2/22	105/18800	0.006636	0.037342	0.020612
CC	GO:0045121	Membrane raft	4/22	326/19594	0.000434	0.007687	0.005317
CC	GO:0098857	Membrane microdomain	4/22	327/19594	0.000439	0.007687	0.005317
CC	GO:0009897	External side of plasma membrane	4/22	455/19594	0.001506	0.013176	0.009114
CC	GO:0001772	Immunological synapse	2/22	44/19594	0.001106	0.012908	0.008929
MF	GO:0005125	Cytokine activity	4/21	235/18410	0.000131	0.004222	0.002894
MF	GO:0048018	Receptor ligand activity	4/21	489/18410	0.002054	0.026583	0.018221
MF	GO:0030546	Signaling receptor activator activity	4/21	496/18410	0.002164	0.026583	0.018221
MF	GO:0005126	Cytokine receptor binding	3/21	272/18410	0.003484	0.033288	0.022817
KEGG	hsa04621	NOD-like receptor signaling pathway	7/17	184/8164	4.24E−08	3.81E−06	2.68E−06
KEGG	hsa04064	NF-κB signaling pathway	5/17	104/8164	1.67E−06	7.51E−05	5.27E−05
KEGG	hsa04668	TNF signaling pathway	4/17	112/8164	6.96E-05	0.002088	0.001465
KEGG	hsa04210	Apoptosis	3/17	136/8164	0.002591	0.02256	0.015831

Abbreviations: BP, biological process; CC, cellular component; GO, Gene Ontology; KEGG, Kyoto Encyclopedia of Genes and Genomes; MF, molecular function.

## Data Availability

The datasets (GSE40611, GSE127952, and GSE154926) used and analyzed in this research are accessible in the NCBI Gene Expression Omnibus (GEO) database: GSE40611 (https://www.ncbi.nlm.nih.gov/geo/query/acc.cgi?acc=GSE40611); GSE127952 (https://www.ncbi.nlm.nih.gov/geo/query/acc.cgi?acc=GSE127952); GSE154926 (https://www.ncbi.nlm.nih.gov/geo/query/acc.cgi?acc=GSE154926). Pyroptosis-related gene sets were obtained from the Molecular Signatures Database (MSigDB; https://www.gseamsigdb.org/gsea/msigdb) and GeneCards (https://www.genecards.org/). miRNA-target interactions were predicted using ENCORI (https://rnasysu.com/encori/). Transcription factors (TFs) were identified using ChIPBase (https://rna.sysu.edu.cn/chipbase/) and hTFtarget (http://bioinfo.life.hust.edu.cn/hTFtarget/). Immune cell infiltration was analyzed using CIBERSORTx (https://cibersortx.stanford.edu/). Functionally similar genes were analyzed using the GeneMANIA prediction server (https://genemania.org/). For further inquiries, please contact the corresponding author.

## Supporting Information

Supporting Information 1 Figure S1. Dataset merging and batch effect correction. Table S1. Complete list of pyroptosis-related genes (PRGs). Table S2. Primer sequences used for RT-qPCR analysis. Table S3. List of predicted miRNA–mRNA interactions for key genes. Table S4. List of predicted TF–mRNA interactions for key genes.

Supporting Information 2 Table S1a. List of pyroptosis-related genes and their respective data sources.
