# Supplementary material for: Identification and Validation of Pyroptosis-Associated Gene Signature in Primary Sjögren's Syndrome
Source: Mediators Inflamm. 2025 Oct 3;2025:1538054. doi: 10.1155/mi/1538054 (PMC12513781; doi:10.1155/mi/1538054)
Supplement: Supporting Information 1 — Figure S1. Dataset merging and batch effect correction. Table S1. Complete list of pyroptosis-related genes (PRGs). Table S2. Primer sequences used for RT-qPCR analysis. Table S3. List of predicted miRNA–mRNA interactions for key genes. Table S4. List of predicted TF–mRNA interactions for key genes. [file 1538054.f1.docx]

## Supplementary figure and tables

##
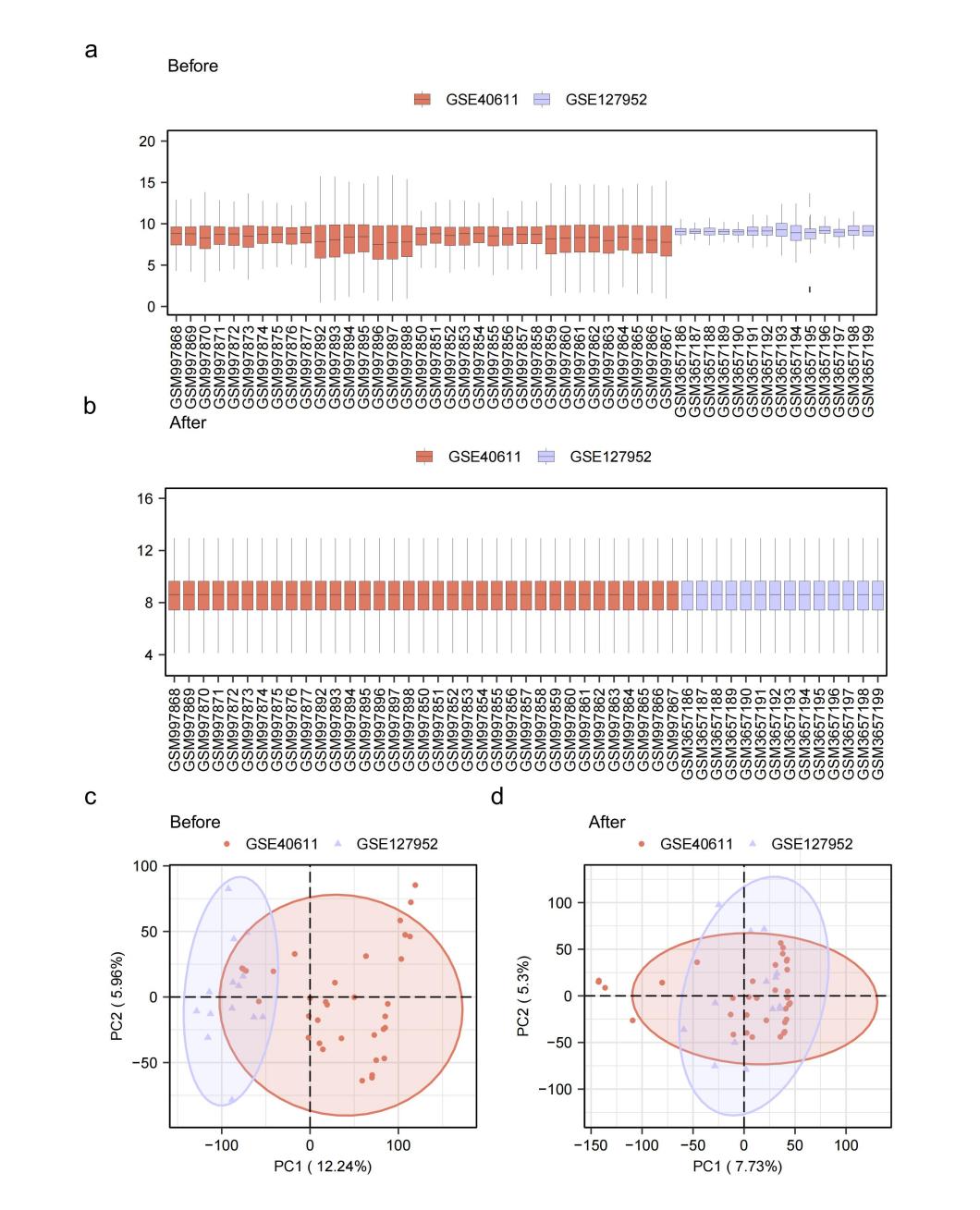


**Figure S1** Dataset merging and batch effect correction. **a** Boxplot showing the distribution of gene expression values in the combined dataset (GSE40611 and GSE127952) before batch effect correction. **b** Boxplot showing the distribution of gene expression values after batch effect correction using the sva package. **c** Principal component analysis (PCA) plot visualizing the combined dataset before batch effect correction, demonstrating clear batch separation. **d** PCA plot after batch effect correction, demonstrating effective removal of batch effects and improved dataset integration. PCA: Principal Component Analysis

## Table S1. Complete list of pyroptosis-related genes (PRGs)

| PRGs |
| --- |
| AIM2 CASP1 CASP3 CASP4 CASP5 CASP6 CASP8 CASP9 ELANE GPX4 GSDMA GSDMB GSDMC GSDMD GSDME IL18 IL1B IL6 NLRC4 NLRP1 NLRP2 NLRP3 NLRP6 NLRP7 NOD1 NOD2 PJVK PLCG1 PRKACA PYCARD TNF BAK1 TP63 CHMP2B BAX GZMB CHMP4B IL1A CHMP3 IRF1 CHMP2A TP53 CHMP7 CHMP4C IRF2 CYCS CHMP6 HMGB1 CHMP4A CARD8 GZMA DPP9 ZBP1 DPP8 TRIM24 NAIP MEFV STAT3 CASP7 SIRT1 DHX9 NLRP9 APIP FOXO3 CTSG NEK7 NFE2L2 GBP1 TREM2 TXNIP FOXP3 SESN2 DDX3X MAPK14 FMR1 GJA1 SNIP1 PRDM1 AGER IL17A PCSK9 PKM VDR BRD4 IKBKE KIF23 IFI16 SEZ6L2 CRTAC1 SMIM1 ETS1 TRAF6 TET2 CEBPB CTSV UTS2 SIGLEC15 ZNF532 MLKL PTPN11 NFKB1 APOE PAK2 ANXA1 P2RX7 SDHB FPR2 CD274 EEF2K HTRA1 ATP6AP1 SETD7 TRIM25 NDUFA13 FGF21 IL37 NLRX1 SYVN1 UBR2 CPTP MMP1 IKZF1 BSG CXCL8 PGF TFAM TRIM21 CEP55 USP25 FAT1 SLC16A4 USF2 IL32 SLC30A7 PIF1 YTHDF2 CHRFAM7A MALT1 TLR2 GSK3B ICAM1 PTGS2 STK4 MST1 PRF1 PRMT5 ATG5 VTN CITED2 PTX3 STING1 ELAVL1 MPEG1 MAP3K20 HDAC6 EPHA2 PPARG ABL1 TLR3 VEGFA SQSTM1 STAT2 CDK9 FGF5 IRF3 IRF9 UCP1 TREM1 TSLP ZDHHC1 PTEN DRD2 ADORA2B ADORA1 ADORA2A ADORA3 ULK1 OSM METTL3 PECAM1 METTL14 S100A12 TRIM31 CAMP MRE11 TRAF3 TFG FNDC4 FNDC5 PARP1 MAP3K7 PRKN USP8 GBP5 NR1H2 MKI67 USP47 TCEA3 IL36G IL36B PRTN3 SERPINB1 DUOX1 APOL1 BNIP3 ANO6 CNR1 FADD VCAM1 CAPN1 VIM JUN RIPK3 MAPK11 CALM1 CALM2 BRCC3 CALM3 BHLHE40 BHLHE41 EZH2 DNMT1 DNMT3A DNMT3B POLA1 RBBP4 EED H2AX PRIM1 RBBP7 H3-3A PRIM2 SUZ12 H2AZ1 POLA2 H2BC21 H3-3B H2AC20 H3C1 H4C3 H2AZ2 H2BC11 H2BC9 H4C11 H2BC14 H3C2 H3C4 H4C1 H4C16 H4C2 H4C8 H4C9 H2BC3 H2BC4 H4C12 H4C5 H2BC1 H2BC13 H2BC15 H2BC5 H3C11 H3C12 H3C14 H4C4 H2AC14 H2AC4 H2AC6 H2AJ H2BC10 H2BC12 H2BC17 H2BC26 H2BC6 H3C10 H3C3 H3C6 H3C7 H3C8 H4C13 H2AC7 H2AC8 H2BC8 H3C15 H4C14 H4C6 H2BC7 H3C13 H2AB1 H2AC18 H4C15 H2AC19 RNF103-CHMP3 LOC102724334 ALK CSNK1A1 CDK1 BIRC2 BIRC3 TFAP2A E2F4 LY96 MIB1 UBE2D2 GLMN SCAF11 SUGT1 IRGM NLRP13 NINJ1 TUBB6 MYD88 TLR8 APAF1 HKDC1 NOS1 NOS2 PKN2 TACR1 DPEP1 TAC1 CHMP1A PYDC2 CCL5 MUC20 ACE2 IL27 EGFR AKT1 ATF6 POP1 ORMDL3 BTK AXL MDM2 HSP90AA1 BCL2 IL1RN HSP90AB1 RIPK1 YWHAE ANXA2 IFIH1 NEDD4 CD14 CSTB HSF1 IRAK3 MELK TLR9 YWHAZ ASIC1 BECN1 GSTO1 HUWE1 NFS1 PANX1 TNFSF13B UBE2D3 CHI3L1 RAB5A STXBP2 ATG7 CDC37 IL13 PDCD6IP USP24 BNIP3L LRPPRC DEPTOR ERP44 GPER1 IL18BP TRPM2 AHSA1 ATG3 VPS4B BST2 BTN3A1 IL13RA2 NCR1 STXBP3 LYST VPS28 CGAS SEC22B CLEC5A SIGLEC14 |

PRGs: Pyroptosis-related genes

## Table S2. Primer sequences used for RT-qPCR analysis

| **LOT, NO.** | **Name** | **Sequence (5' → 3')** |
| --- | --- | --- |
| A2312080056 | CRTAC1-F(mus) | TGGCAGTGACAGATGTGGAC |
| A2312080057 | CRTAC1-R(mus) | ACGGTCCCTCAGAGCATAGT |
| A2312080058 | PECAM1-F(mus) | CACACCGAGAGCTACGTCAT |
| A2312080059 | PECAM1-R(mus) | TTGGATACGCCATGCACCTT |
| A2312080060 | Irf2-F(mus) | TCTCCTGAGTATGCGGTCCT |
| A2312080061 | Irf2-R(mus) | ATGTCTGGCGGGTTAGTGAC |
| A2312080062 | GZMA-F(mus) | ACACGGTTGTTCCTCACTCA |
| A2312080063 | GZMA-R(mus) | AGCAGTCAACACCCAGTTCT |
| A2312080064 | IFI16-F(mus) | TGGCACAACATCAACTGCAAG |
| A2312080065 | IFI16-R(mus) | GCACCATCACTTGTTTGGGAC |
| A2312080066 | AIM2-F(mus) | AGACTCAGGAAGGAAGACAAGA |
| A2312080067 | AIM2-R(mus) | GGTGACCTCCATGAAGTTACTG |
| A2312080068 | TNF-F(mus) | GCCGATGGGTTGTACCTTGT |
| A2312080069 | TNF-R(mus) | TCTTGACGGCAGAGAGGAGG |
| A2312080070 | MPEG1-F(mus) | CAACTCACGGGTGCAGAGTT |
| A2312080071 | MPEG1-R(mus) | GCCAGGTAGCTTGTCAGGTT |
| A2312080072 | GAPDH-F(mus) | TGGAAAGCTGTGGCGTGATG |
| A2312080073 | GAPDH-R(mus) | TACTTGGCAGGTTTCTCCAGG |

## Table S3. List of predicted miRNA-mRNA interactions for key genes

| miRNA | mRNA |
| --- | --- |
| hsa-miR-382-5p | IFI16 |
| hsa-miR-18a-5p | IRF2 |
| hsa-miR-23a-3p | IRF2 |
| hsa-miR-214-3p | IRF2 |
| hsa-miR-221-3p | IRF2 |
| hsa-miR-222-3p | IRF2 |
| hsa-miR-23b-3p | IRF2 |
| hsa-miR-133a-3p | IRF2 |
| hsa-miR-153-3p | IRF2 |
| hsa-miR-320a | IRF2 |
| hsa-miR-320a | IRF2 |
| hsa-miR-302a-3p | IRF2 |
| hsa-miR-302b-3p | IRF2 |
| hsa-miR-302c-3p | IRF2 |
| hsa-miR-302d-3p | IRF2 |
| hsa-miR-372-3p | IRF2 |
| hsa-miR-373-3p | IRF2 |
| hsa-miR-133b | IRF2 |
| hsa-miR-18b-5p | IRF2 |
| hsa-miR-448 | IRF2 |
| hsa-miR-485-5p | IRF2 |
| hsa-miR-496 | IRF2 |
| hsa-miR-520e | IRF2 |
| hsa-miR-520f-3p | IRF2 |
| hsa-miR-520f-3p | IRF2 |
| hsa-miR-520a-3p | IRF2 |
| hsa-miR-520b | IRF2 |
| hsa-miR-520c-3p | IRF2 |
| hsa-miR-524-5p | IRF2 |
| hsa-miR-520d-5p | IRF2 |
| hsa-miR-520d-3p | IRF2 |
| hsa-miR-455-5p | IRF2 |
| hsa-miR-556-5p | IRF2 |
| hsa-miR-579-3p | IRF2 |
| hsa-miR-770-5p | IRF2 |
| hsa-miR-130a-5p | IRF2 |
| hsa-miR-340-5p | IRF2 |
| hsa-miR-340-5p | IRF2 |
| hsa-miR-513b-5p | IRF2 |
| hsa-miR-513b-5p | IRF2 |
| hsa-miR-320b | IRF2 |
| hsa-miR-320b | IRF2 |
| hsa-miR-320c | IRF2 |
| hsa-miR-320c | IRF2 |
| hsa-miR-302e | IRF2 |
| hsa-miR-320d | IRF2 |
| hsa-miR-320d | IRF2 |
| hsa-miR-1193 | IRF2 |
| hsa-miR-1193 | IRF2 |
| hsa-miR-1193 | IRF2 |
| hsa-miR-3200-3p | IRF2 |
| hsa-miR-4735-3p | IRF2 |
| hsa-miR-29a-3p | MPEG1 |
| hsa-miR-212-3p | MPEG1 |
| hsa-miR-132-3p | MPEG1 |
| hsa-miR-194-5p | MPEG1 |
| hsa-miR-155-5p | MPEG1 |
| hsa-miR-155-5p | MPEG1 |
| hsa-miR-34c-5p | MPEG1 |
| hsa-miR-449b-5p | MPEG1 |
| hsa-miR-181a-5p | TNF |
| hsa-miR-181b-5p | TNF |
| hsa-miR-181c-5p | TNF |
| hsa-miR-130a-3p | TNF |
| hsa-miR-301a-3p | TNF |
| hsa-miR-130b-3p | TNF |
| hsa-miR-181d-5p | TNF |
| hsa-miR-454-3p | TNF |
| hsa-miR-301b-3p | TNF |

## Table S4. List of predicted TF-mRNA interactions for key genes

| TF | mRNA |
| --- | --- |
| BRD4 | CRTAC1 |
| EGR1 | CRTAC1 |
| EP300 | CRTAC1 |
| JUN | CRTAC1 |
| JUND | CRTAC1 |
| KLF4 | CRTAC1 |
| LMNB1 | CRTAC1 |
| MAZ | CRTAC1 |
| MYOD1 | CRTAC1 |
| NR2F2 | CRTAC1 |
| SPI1 | CRTAC1 |
| TCF12 | CRTAC1 |
| TFAP2C | CRTAC1 |
| ZBTB7A | CRTAC1 |
| JUN | CRTAC1 |
| JUND | CRTAC1 |
| KLF4 | CRTAC1 |
| MAZ | CRTAC1 |
| MYOD1 | CRTAC1 |
| NR2F2 | CRTAC1 |
| BRD4 | CRTAC1 |
| RUNX2 | CRTAC1 |
| SPI1 | CRTAC1 |
| TCF12 | CRTAC1 |
| TFAP2C | CRTAC1 |
| ZBTB7A | CRTAC1 |
| EP300 | CRTAC1 |
| ETS1 | CRTAC1 |
| FOXA1 | CRTAC1 |
| EP300 | IFI16 |
| ERG | IFI16 |
| ETS1 | IFI16 |
| ATF2 | IFI16 |
| FLI1 | IFI16 |
| FOS | IFI16 |
| FOSL1 | IFI16 |
| FOXA2 | IFI16 |
| ATF3 | IFI16 |
| GATA1 | IFI16 |
| HDAC1 | IFI16 |
| HDAC2 | IFI16 |
| IRF1 | IFI16 |
| IRF4 | IFI16 |
| JUN | IFI16 |
| KLF4 | IFI16 |
| MTA3 | IFI16 |
| MYB | IFI16 |
| BRD2 | IFI16 |
| NFIC | IFI16 |
| BRD4 | IFI16 |
| RAD21 | IFI16 |
| SMC1A | IFI16 |
| SPI1 | IFI16 |
| CDK9 | IFI16 |
| STAT1 | IFI16 |
| CEBPA | IFI16 |
| CEBPB | IFI16 |
| CREBBP | IFI16 |
| ARNT | IFI16 |
| EBF1 | IFI16 |
| E2F1 | IRF2 |
| E2F6 | IRF2 |
| ELK3 | IRF2 |
| EP300 | IRF2 |
| ERG | IRF2 |
| ETS1 | IRF2 |
| FLI1 | IRF2 |
| FOXA1 | IRF2 |
| FOXA2 | IRF2 |
| FOXP2 | IRF2 |
| GABPA | IRF2 |
| GATA1 | IRF2 |
| ATF3 | IRF2 |
| HDAC1 | IRF2 |
| HDAC2 | IRF2 |
| HNF4G | IRF2 |
| IRF1 | IRF2 |
| IRF4 | IRF2 |
| KLF1 | IRF2 |
| KLF5 | IRF2 |
| KMT2A | IRF2 |
| LMNB1 | IRF2 |
| LMO2 | IRF2 |
| MAX | IRF2 |
| MAZ | IRF2 |
| MEF2A | IRF2 |
| BCL6 | IRF2 |
| BCOR | IRF2 |
| NFIC | IRF2 |
| NFYB | IRF2 |
| NR2F2 | IRF2 |
| ONECUT1 | IRF2 |
| PAX5 | IRF2 |
| PBX3 | IRF2 |
| BRD4 | IRF2 |
| PPARG | IRF2 |
| RUNX1T1 | IRF2 |
| RUNX3 | IRF2 |
| RXRG | IRF2 |
| SPI1 | IRF2 |
| SPIB | IRF2 |
| STAG1 | IRF2 |
| TCF3 | IRF2 |
| TFAP4 | IRF2 |
| WDR5 | IRF2 |
| CDK8 | IRF2 |
| CDK9 | IRF2 |
| ZBTB7A | IRF2 |
| CEBPA | IRF2 |
| CEBPB | IRF2 |
| CEBPD | IRF2 |
| ZNF263 | IRF2 |
| CREBBP | IRF2 |
| DDX5 | IRF2 |
| IRF4 | MPEG1 |
| KLF4 | MPEG1 |
| MEF2A | MPEG1 |
| NCOR2 | MPEG1 |
| PAX5 | MPEG1 |
| POU2F2 | MPEG1 |
| RAD21 | MPEG1 |
| RUNX3 | MPEG1 |
| RXRG | MPEG1 |
| SMC1A | MPEG1 |
| SMC3 | MPEG1 |
| SPI1 | MPEG1 |
| SRF | MPEG1 |
| STAG1 | MPEG1 |
| TCF3 | MPEG1 |
| TFAP4 | MPEG1 |
| YY1 | MPEG1 |
| ATF2 | MPEG1 |
| BATF | MPEG1 |
| BCL11A | MPEG1 |
| BCL6 | MPEG1 |
| CDK9 | MPEG1 |
| CEBPA | MPEG1 |
| CEBPB | MPEG1 |
| CTCF | MPEG1 |
| EBF1 | MPEG1 |
| FLI1 | MPEG1 |
| MAZ | TNF |
| SPI1 | TNF |
| TCF12 | TNF |
| TCF3 | TNF |
| CDK9 | TNF |
| CTCF | TNF |
| EBF1 | TNF |
| ERG | TNF |
| ETS1 | TNF |
| FLI1 | TNF |
| GATA1 | TNF |
| GATA3 | TNF |
